# Smart polymers for cell therapy and precision medicine

**DOI:** 10.1186/s12929-019-0571-4

**Published:** 2019-10-18

**Authors:** Hung-Jin Huang, Yu-Liang Tsai, Shih-Ho Lin, Shan-hui Hsu

**Affiliations:** 1 Institute of Polymer Science and Engineering, National Taiwan University, No. 1, Sec. 4 Roosevelt Road, Taipei, 10617 Taiwan Republic of China; 2 Research and Development Center for Medical Devices, National Taiwan University, Taipei, Taiwan; 3 Institute of Cellular and System Medicine, National Health Research Institutes, No. 35 Keyan Road, Miaoli, 35053 Taiwan Republic of China

**Keywords:** Smart materials, Bioprinting, Cell therapy, Tissue engineering, Precision medicine

## Abstract

Soft materials have been developed very rapidly in the biomedical field over the past 10 years because of advances in medical devices, cell therapy, and 3D printing for precision medicine. Smart polymers are one category of soft materials that respond to environmental changes. One typical example is the thermally-responsive polymers, which are widely used as cell carriers and in 3D printing. Self-healing polymers are one type of smart polymers that have the capacity to recover the structure after repeated damages and are often injectable through needles. Shape memory polymers are another type with the ability to memorize their original shape. These smart polymers can be used as cell/drug/protein carriers. Their injectability and shape memory performance allow them to be applied in bioprinting, minimally invasive surgery, and precision medicine. This review will describe the general materials design, characterization, as well as the current progresses and challenges of these smart polymers.

## Background

Soft materials with similar shear modulus or mechanical strength to human body tissues have been highly focused in the decades, especially those soft materials with unique properties that we called “smart polymeric materials”. Since the concept of one-size-fits-all was outdated, scientists and engineers have designed smart materials into tunable and personalized products to overcome many limitations during the heterogeneous environment in the human body. Smart materials, also known as responsive materials, are synthetic materials with one or more properties that can be significantly altered in a controlled manner by external stimuli [1]. Polymeric smart materials are most commonly used in the biomedical field, owing to not only introducing the high biocompatibility from natural polymers [2] but also the tunable and functional properties from synthetic polymers [1]. There are many external stimuli for smart materials, including temperature [3], redox reactions [4], humidity [5], electric or magnetic field [6], pH changes [7], and light intensity [8]. Those materials with different triggering mechanisms were used for various biomedical applications, including biosensors [9], controllable drug delivery [10, 11], tissue repairing [12], local injection, cancer cell separators, minimally invasive surgery, and 3D bioprinting [13], etc. The tunable properties and environmental responses of the smart polymeric materials provide the opportunity to design personalized biomedical products. This review paper will emphasize three kinds of typical polymeric smart materials, including stimulus-responsive, self-healing, and shape memory in materials. The review also discusses some recent applications in precision medicine, such as 3D bioprinting, cell therapy, and tissue engineering.

Stimulus-responsive materials have been employed to develop novel medical devices for minimally invasive procedures. For instance, thermoresponsive and pH-sensitive hydrogels are commonly used for cardiac therapies at low local pH of an infarcted area [14]. By exposing to stimuli, the responsive polymeric materials show a local response, which can be a trigger for intracellular drug delivery for tissue targeting [15]. Moreover, self-healing hydrogels are 3D chemical or physical reversible network that can recover the original morphology after damages. Dynamic linkages dominate the dissociation and recombination processes and endow the self-healing property similar to human tissue repairing [16]. Therefore, self-healing hydrogels with appropriate viscoelasticity and high water contents are employed to mimic the extracellular matrix for providing a suitable environment for cell culture. Self-healing hydrogels have achieved significant success in central nervous rescued, vascular tissue reconstruction, and the anti-cancer drug delivery, etc. [17–19]. In addition, shape memory materials (SMMs) are commonly divided into two groups, shape memory alloys (SMAs) and shape memory polymers (SMPs). After being severely and quasi-plastically distorted [20], SMMs can recover to their original shapes with proper stimuli. SMAs are usually utilized for cardiovascular stents or orthodontic devices [21], whereas SMPs can be used in cardiac valves, kidney dialysis, and neuroprosthetics [22]. SMPs with biocompatibility and biodegradability are potential materials for many advanced applications, such as soft robotics, artificial skin, and 4D printing in the future [23–25].

Precision medicine, also called personalized medicine, is an avant-garde field of health care that relies on a person’s clinical, genetic, and environmental conditions. To realize precision medicine, it needs to integrate a wide range of fields, including smart biomaterials, pathology, large-scale biological databases, and medical devices [26]. The development of smart polymeric materials that exhibit good biocompatibility and adjustability may accelerate the progress of precision medicine because the properties of smart polymeric materials can be designed to own properties highly specific for individuals [14].

**Fig. 1:**
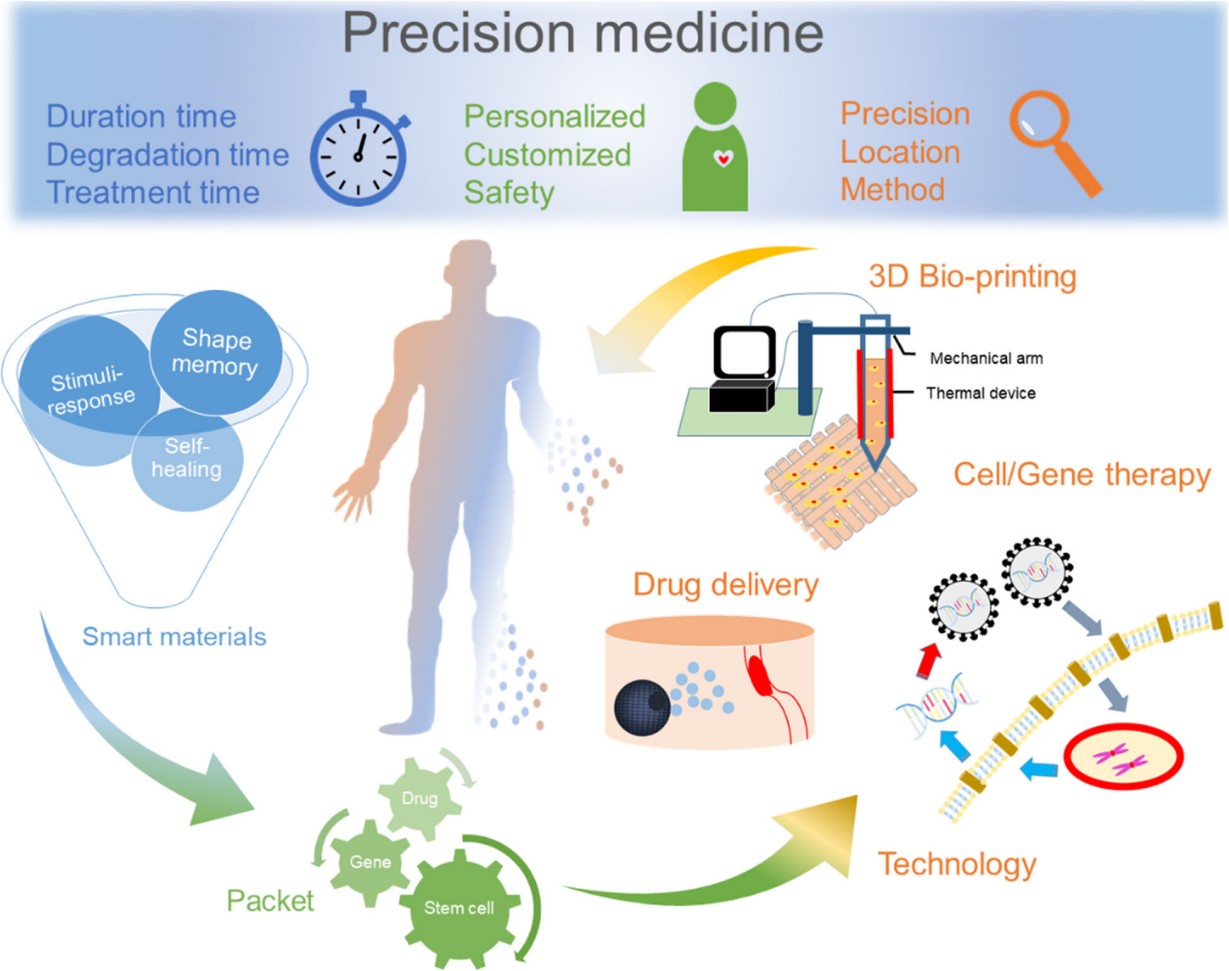
An overview of the process diagram of precision medicine, including three core concepts, the activation time, customized design, and local delivery

Three core values of precision medicine are compiled for the relationship between precision medicine and materials in Fig. 1, “temporal”, “personal”, and “spatial”. The temporal perspective concerns the balance between the treatment time of the patients and the degradation time of the smart polymeric materials. The polymeric materials for in vivo applications, such as drug carriers, wound dressing, and bioglue [27], are either degradable or absorbable after the treatment, and the degradation products should not be toxic or harmful. From the personal perspective, the smart polymeric materials should be capable of carrying patients’ stem cells or genes for cell/gene therapy [28]. From the spatial perspective, the materials should respond to particular stimuli and release drugs at designed locations. To fulfill these three perspectives, the combination of technology and materials is highly demanded. For example, 3D bioprinting of shape memory materials is one good strategy to prepare customized biomedical devices, and surgeons will be able to utilize the patient-orientated product to perform minimally invasive surgery [15]. In this review, several smart materials will be introduced in the aspects of limitations and biomedical applications.

## Smart polymeric materials in biomedicine

Smart polymeric materials with stimuli-responsive, self-healing, and shape memory behaviors can be applied in the biomedical field. The combination of the three features is beneficial in precision medicine. The important concepts and the advantages of smart polymeric materials with three categories in precision medicine are summarized in Table 1. In the following sections, we summarize the compositions, mechanisms, and applications of different types of smart polymeric materials.

**Table 1:** The mechanisms, components, and applications of smart polymers in precision medicine

Category	Mechanism	Component	Applications	Benefit	References
Stimuli response	Temperature	NiPAAm	Biomolecule carriers	Injectable	[29]
			Wound dressing	Long-term antimicrobial and anti-protein absorption	[30]
			Sensing, imaging, and carrier	Multifunctional sensing and imaging	[31]
			Cell culture platform	Bioactive cell recovery	[32]
		PU	Cell or drug carrier	Highly tunable	[33]
			Neural tissue engineering	Printable bioink	[34]
		Pluronic	Drug delivery	Injectable, thermo-responsive	[35]
	Photo	Epoxy resins	Dental restorative or fillers	Durable, easy operation	[36]
		GelMA	Cell culture platform	Printable multi cells	[37]
			Cartilage tissue engineering	Animal model	[38]
			Bone tissue engineering	Structurally stable for large bone defects	[39]
		PU	Neural tissue engineering	Printable soft bioink	[8]
Self-healing	Physical interaction	Poly(styrene-acrylic acid)	Artificial cartilage or skin	High mechanical strength	[40, 41]
		Poly(glyceryl amine)	Dermal drug delivery	Strong penetration ability	[42]
		Polyaniline, phytic acid	Wearable electronics	Mechanically robust	[43]
		Silver-nucleoside complex [Ag(I)-(N3-cytidine)2]	Metallo-DNA	Thixotropic self-healing	[44]
	Chemical covalent bond	Polyurea, HA	Polyurea flooring systems	Crack repair	[45]
		HA, glycol chitosan	Cartilage tissue engineering	Biocompatibility	[46]
		Graphene nanoplate	Electronic devices	Electrical conductivity	[47]
Shape memory	Temperature	PU	Shape memory stents or scaffolds	Biodegradability	[48]
		N,N-dimethylacrylamide	Artificial intervertebral disk	Strong interface	[49]
	Water	PU	Bone tissue engineering	Printable bioink	[50]
		Cellulose	Pressure sensor	Zero poisson ratio, durable	[51]
	pH	Alginate	Bioglue	Adhesive	[52]

### Responsive (thermal / photo) polymers: Thermoresponsive polymers

Pluronics, also known as Poloxamers, are nonionic triblock copolymers consisting of a central hydrophobic polypropylene oxide (PPO) block flanked by hydrophilic polyethylene oxide (PEO) blocks. Due to the hydrophobic interaction, Pluronics self-assemble in the aqueous solution, and the micellization conditions of Pluronics are dominated by both concentration and temperature [53, 54]. The hydrophobic segments of Pluronic aggregate in order to minimize surface tension when concentration is higher the critical micelle concentration (CMC) and the temperature is lower critical solution temperature (LCST) [55, 56]. The amphiphilic polymers also show some weaknesses such as rapid dissolution, short residence time, and weak mechanical strength [57]. To solve the problems, the modification of hydroxyl groups at the end of the chain provides a good opportunity to overcome the previous mentioned disadvantages [57]. Pluronics with different ratios of bioinert PEO and PPO segments (L121, P123, F127, etc.) show low foreign body reactions and have tunable properties for customized precision medicine. Taken together, the Pluronic and its derivatives display wide-range usages such as tissue regeneration scaffolds [58], antineoplastic delivery [59], super-tough hydrogel [60], and antibacterial adhesive [61].

Poly (N-isopropyl acrylamide) (PNiPAAm) is a type of thermosensitive polymers that undergoes a hydrophilic-hydrophobic transition in water at the LCST around 32 °C. The PNiPAAm demonstrates hydrophilic behavior for hydrogen bonds between water molecules and amide groups when the temperature is below the LCST. When the temperature is above LCST, PNiPAAm becomes hydrophobic since water molecules are expelled from the hydrophilic region of PNiPAAm [29, 30]. With the thermoresponsiveness between room temperature and physiological temperatures, PNiPAAm has been employed to design a variety of smart biomaterials, such as drug or cell delivery vehicles, imaging or sensing, fiber mats for cell recovery, and strain sensors [32, 62].

Polyurethane nanoparticles (PU NPs) with different compositions of biodegradable oligodiols as the soft segment have been reported to be thermoresponsive. The morphological change and rheological behavior of PU NPs are thermal dependents because of different degrees of crystallinity and strength of hydrogen bonding in soft segment compositions. With the thermoresponsive feature, PU NPs can be designed as cell delivery vehicles, biodegradable stents, or bioinks [33, 34, 48].

### Responsive (thermal / photo) polymers: Photoresponsive polymers

Photoresponsive polymers, the light-mediated materials, benefit from precisely spatiotemporal tunability. The common photochemical reactions include bond formation, cleavage, isomerization, and molecular rearrangement [63]. The mechanical properties of photoresponsive polymers can be manipulated by different types of light sources, light dosages, and photoinitiators. Photoinitiators, which are widely-used in bioengineering applications, are Irgacure 2959 (2-Hydroxy-1-(4-(2-hydroxyethoxy)phenyl)-2-methyl-1-propano), LAP (lithium phenyl-2,4,6-trimethylbenzoylphosphinate), eosin-Y, and VA-086 (2,2’-Azobis[2-methyl-N-(2-hydroxyethyl)propionamide]) due to low cytotoxicity, and water solubility [64–66]. The exposure time and the intensity of photo-triggered process, which can be precisely controlled, are two major advantages of using photoresponsive polymers as biomaterials [67, 68]. Epoxy resins were one of the earliest photoresponsive polymers in dental applications, namely, dental restoratives and fillers. The resins allowed in-situ polymerization and on-site treatments [36, 69]. Acylate-based monomers undergo radical polymerization through ultraviolet (UV) light exposure to produce thin films for biomedical applications [68]. O-nitrobenzyl succinate and disulfide were photosensitive moieties, which were utilized to facilitate the synthesis of micelles and particles for controlled drug delivery vehicles [70, 71]. A double-threaded rotaxane dimer with α-cyclodextrin and stilbene was an example of photo-triggered moieties that could potentially lead to the development of artificial muscles [72]. A photosensitive PU hydrogel was also reported to be a promising candidate for 3D bioprinting, particularly in the field of neural tissue engineering [8]. Gelatin methacryloyl (GelMA) was a widely used semisynthetic biomaterial with gelatin and methacryloyl moieties, which rendered GelMA photocrosslinkable by UV light or visible light [37, 73]. Different cells could be loaded in GelMA hydrogels for tissue engineering or 3D bioprinting [74–76]. In addition, GelMA cryogels were fabricated as a matrix for tissue engineering and trigger-release cell therapy [38, 77]. GelMA is one of the most popular photoresponsive biomaterials because of convenient synthesis, biocompatibility, printability, and the translational results from in vivo experiments to realize cell therapy and precision medicine.

### Self-healing polymers

Self-healing polymers are one classic type of smart polymers that can recover the structure after repeated damages and restoring the original functionality [13]. Self-healing hydrogels are of particular interest because of high water contents and controllable rheological properties [78, 79]. With the as mentioned features, self-healing hydrogels mimic extracellular matrix, making this class of smart polymers competitive candidates for biomedical applications [16].

**Fig. 2:**
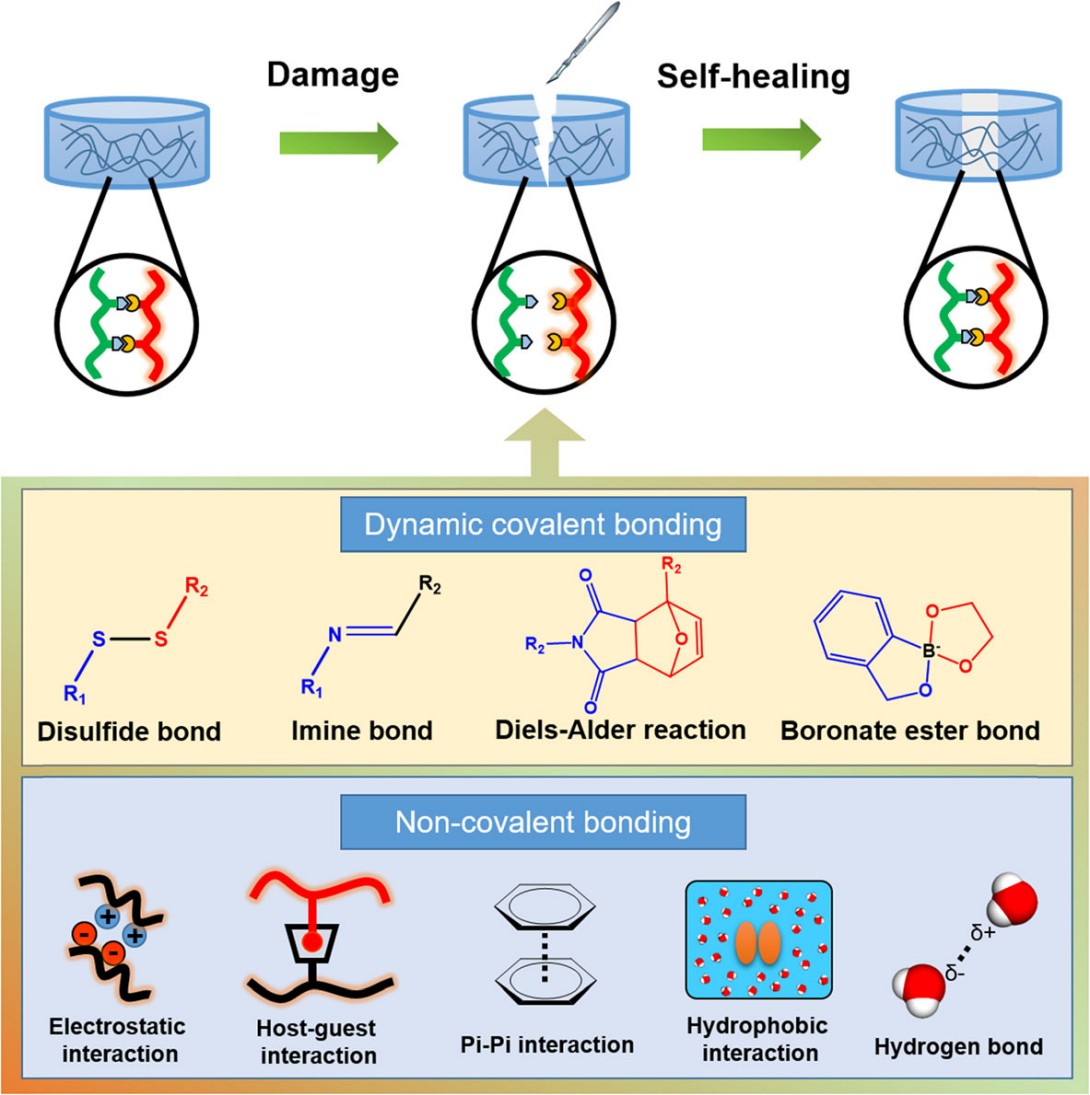
Categories and healing mechanism of self-healing hydrogels for the design strategies of biomedical applications

Two mechanisms of self-healing hydrogels are proposed to explain the dynamic and reversible bonding, shown in Fig. 2. They are non-covalent interactions and dynamic covalent bonds. Non-covalent interactions include hydrogen bond [40], host-guest interactions [42], electrostatic interactions [43], π-π interactions [80], and hydrophobic interactions [44]. The weak intermolecular forces of non-covalent interactions facilitate the reversible assembly and disassembly of self-healing hydrogels. Dynamic covalent bonds include disulfide bonds [45], imine bonds [46], boronate ester bonds [81], and Diels-Alder reactions [47].

The rheological properties of self-healing hydrogels can be specifically tuned, and the hydrogels that possess shear-thinning behavior are injectable. After being injected via needles, hydrogels remain their structures; thus, It is possible to load patients’ cells in the hydrogels for cell therapy and precision medicine [82]. Additionally, self-healing hydrogels were reported to be wound dressings, strain sensors, cell/drug/protein carriers, and bioelectronics devices [83–89]. Chitosan-based and hyaluronic acid-based hydrogels attract the most attention in self-healing hydrogels because of their biocompatibility and biodegradability. Moreover, they can be introduced with special functional groups and have been successfully applied in many biomedical applications [61, 86, 90].

### Shape memory polymers

**Fig. 3:**
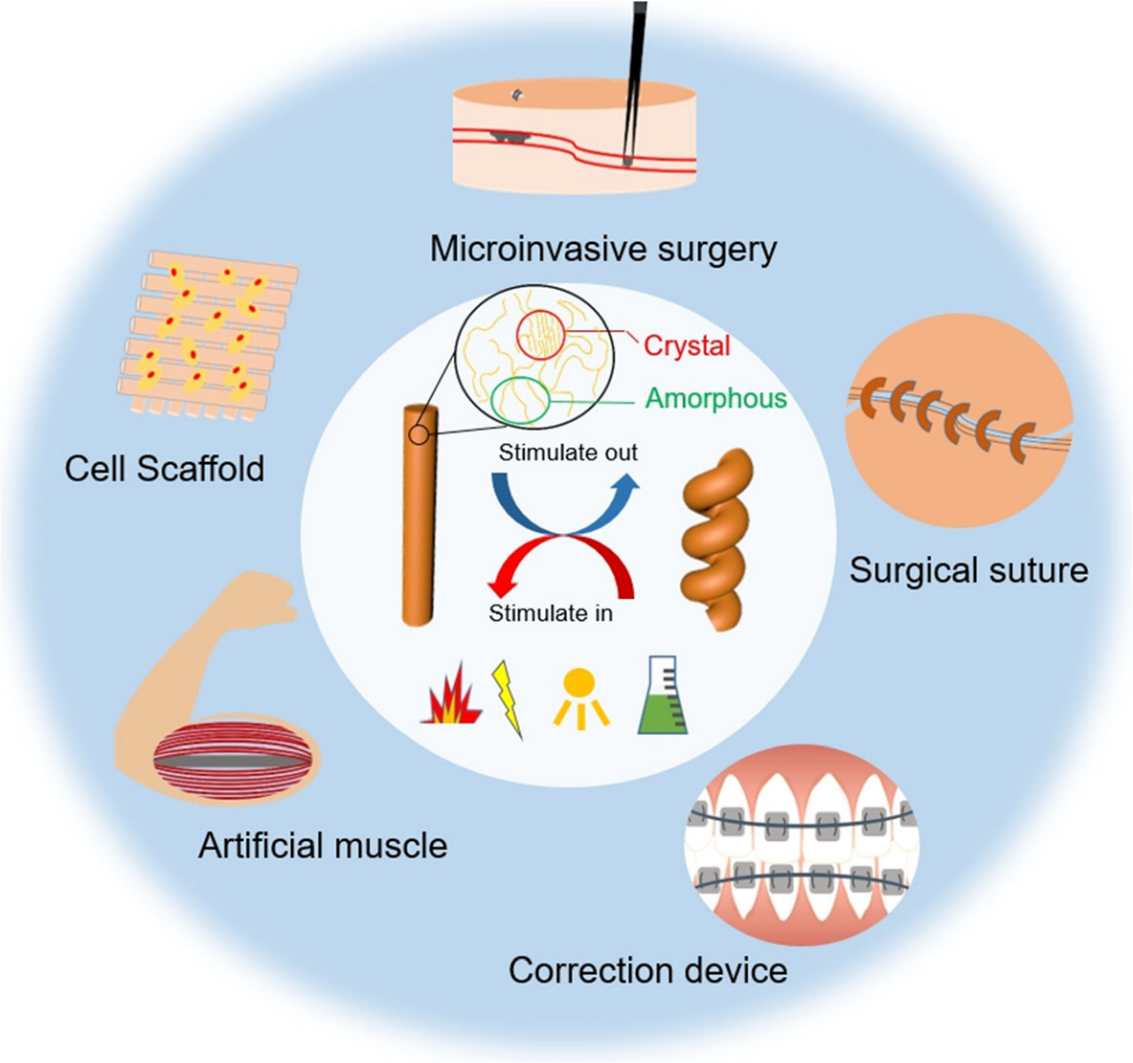
Schematic overview of shape memory materials, including mechanisms, sources of stimulation, various practical or potential applications

SMPs are polymeric materials that can temporarily fix one or more shapes and later recover to the original shape in response to external stimuli, such as heat, chemicals, pH value, or light [15]. The shape memory behavior of SMPs is commonly triggered by supramolecular interactions or dynamic covalent bonds. The potential applications of SMPs are displayed in Fig. 3. SMPs undergo uncoupling and recoupling of non-covalent interactions via supramolecular interactions, including hydrogen bonds, host-guest interactions, and metal-ligand coordination. Meanwhile, SMPs rely on dissociation and recombination of dynamic covalent linkages, including boronate ester bond, imine bond, and disulfide bond [16, 91, 92]. Among SMPs, the ones that react to external responsive under physiological conditions have potentials in biomedical applications. The cellular-structured nanofibrous hydrogel was reported to have shape memory ability triggered by water molecules [51]. Boronate ester hydrogel could be designed to have shape memory ability via pH value variation [52]. The hydrogel composed of N,N-dimethylacrylamide and other acrylate moieties was reported to have the capability of shape memory through exposing to UV light [49]. In previous researches, PU NPs with different oligodiols as the soft segment possess thermal induced shape memory behavior [48, 50]. Briefly, PU is one of the most competitive shape memory biomaterials for cell therapy and precision medicine owing to the biocompatibility and possibility to print.

## Applications of smart polymers for precision medicine

Smart polymers with tailorable mechanical strength, precise shapes, and environmental responsiveness can be employed to produce scaffolds or stents to provide niche to cater with different types of cells for cell therapy. Smart polymers that possess stimulus-responsiveness can be designed as trigger-release cell/drug/protein carriers. Additive manufacturing (AM) is one fabricating strategy to shorten the distance between materials and cell therapy [93]. Therefore, the following sections will include medical devices, cell therapy, and 3D bioprinting as promising tools to facilitate the realization of precision medicine.

### Medical devices

Medical devices are the devices intended to be used for medical purposes. Among them, scaffolds have been widely studied in decades. The main purpose of fabricating scaffolds is to mimic the extracellular matrix or replace damaged tissues or organs [94]. Many significant successes have already been achieved in almost every tissue, such as heart valves [95], brain [93], retina [96], tracheal tissue [97] and skin [98]. Cell/drug/protein carriers are other typical medical devices for precision medicine. Submicron-sized colloidal particles [99], block copolymer micelle [100], and liposome [101] have been developed as drug vehicles, but they sometimes show limitations in rapid and undesired release or diffusion barrier. One of the most important challenges in medical devices is further enhancing the performance of the scaffold by incorporating smart polymers with stimuli response [102]. The introduction of smart polymers makes these medical devices versatile in biomedical applications. For example, flexible and biodegradable shape memory scaffolds were used for a cardiac patch [102], and stimuli-responsive drug carriers allowed the temporal or spatial control of drug release in the diseased tissues [103]. Moreover, these smart devices are expected to have more advanced applications, such as biomimetic 4D printing [104] or self-folding machines [105]. The shortcomings of these devices are often limited by the precise control, cycles of repetition, and the balance between durability and degradation. In general, smart materials demonstrate the potential for fabricating customized medical devices.

### Cell therapy

Cell therapy is considered as a promising therapeutic approach in regenerative medicine, which based on utilizing stem, primary or progenitor cells to facilitate the regeneration of damaged tissue or organs. Current cell-based therapy is designed for various prominent disorders, and diseases targeted by cell-based therapies including cardiovascular, neurological, ophthalmologic, skeletal, autoimmune, and so on [106]. Stem cells can differentiate into specifically functional cell types, which have great capability for tissue regeneration and repair in the human body [107, 108]. Hence, stem cell therapy has emerged as a possible therapeutic strategy because of its inherent self-renewal potential [109]. However, clinical applications of ESCs have ethical challenges and safety concerns [110]. The human embryonic stem cell (hESC) researches contain the destruction of a human embryo, which set a limitation in developing stem cell therapy based on hESC. Besides, immunologic rejection [111] and teratoma formation [112] have safety concerns for the clinical application of stem cells. The induced pluripotent stem cells (iPSCs) technology can avoid the destruction of human embryos and bypass ethical problems, which offers an opportunity to realize precision and personalized medicine [113, 114].

**Fig. 4:**
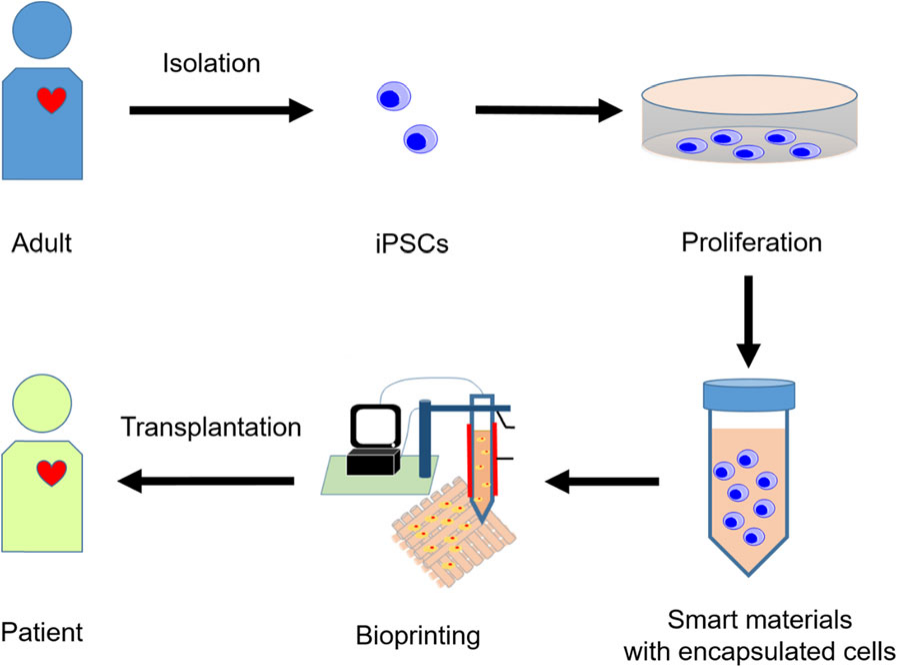
The workflow of smart materials in combination with stem cells for therapeutic applications through bioprinting process

Smart materials can be customized and designed in combination with stem cells and bioprinting. The diagram of transplantation and cell therapy is shown in Fig. 4. The strategy of 3D bioprinting integrates smart materials and stem cells to control cell proliferation or differentiation. Smart materials can change their properties via external stimulations that have the potential to fulfill stem cell differentiation over the past decade [115, 116]. The application of smart materials has been utilized to regulate the differentiation of the stem cells to target cells in response to various external stimulations in recent years [117]. The proliferation of stem cells under the specific biological environment conditions can be applied to develop clinical approaches for regeneration and repair of damaged tissues, including cartilage, bone, nerves, fat, and muscle.

Precision biomaterials can be defined as biomaterials designed to target various diseases for individual patients [26]. Customized biomaterials might face challenges that hinder the advancement of designing precision biomaterials such as clinical usages, state of disease, practicality, cost, and approval for use [26]. For instance, the design of smart materials that have the ability to control cellular behavior is a significant challenge in local delivery systems [118]. The lack of biodegradability and biocompatibility might cause adverse effects in strategies of tailored treatment. After implantation of the designed materials to individuals, unwanted inflammation is the major concern that hinders the role of biomimetic approach in the process of precision treatments [119, 120].

One of the major challenges is to control the differentiation of stem cells to specific cell types. For instance, recent studies displayed that chitosan-based substrates are effective methods to drive self-renewal, self-assembly, and differentiation of stem cells [121, 122]. By tuning the properties of smart materials, it is possible to regulate the differentiation of stem cells serving as a therapeutic platform for cell-based therapy. Designing smart materials can help stem cells differentiate into specific tissue for tissue regeneration and personalized medicine [123]. Smart materials with self-healing ability can be used in cell printing, which may influence the differentiation of iPSCs to target cells and allow more convenient approach to replace damaged tissue or for applications in minimally invasive surgery [124].

### 3D printing

AM, commonly known as 3D printing, is a promising technique to fabricate customized shapes or replicate human-scale tissues [82, 125]. Extrusion-based 3D printing is an important tool to perform 3D bioprinting, and this technique is feasible to incorporate high cell density in the aforementioned smart materials [126]. The printing parameters rely on the shear-thinning property of the bioink, and the bioink needs to protect the embedded cells when it goes through the nozzle. The crosslinking mechanisms and mechanical properties of bioink also play essential roles in cell viability [127–129]. Collagen and agarose-based bioinks seeded with stem cells are reported to maintain cell viability and induce cell differentiation [130]. GelMA based bioinks show good printability and promote cell proliferation. The mechanical properties of GelMA based bioinks could be tuned based on different crosslinking density, exposure time, and light sources, UV light or visible light [73–75]. PU NP bioink also reveals good printability and an appropriate 3D environment for cell culture [8].

Previous studies suggested that bioink can be a versatile platform for different tissue engineering [34, 50, 97, 131, 132]. In addition to bioink, sacrificial 3D printing is an alternative strategy to provide hollow tube constructs for tissue engineering. In the previous studies, the sacrificial materials could be removed due to their environmental responsiveness, including temperature variation and chemical dissolution [133–135]. Moreover, stereolithography (SLA) is another type of 3D printing, which uses light to selectively trigger the polymerization of photopolymers. Synthetic biomaterials, such as poly (D,L-lactide), poly(ethylene glycol) diacrylate, and poly(ɛ-caprolactone) have been 3D printed via SLA to fabricate scaffolds with high resolution.

## Conclusion

The stimulus-responsive property, shape memory behavior, and self-healing ability of smart polymeric materials are important features for tissue engineering, medical devices, and cell therapy. Precision medicine can be gradually realized with smart polymeric materials considering the “temporal”, “spatial”, and “personal” aspects. Smart polymeric materials are promising biomedical materials for the development of novel biodegradable or biocompatible scaffolds to fulfill the temporal aspect. Designing smart polymeric materials also helps improve the development of injectable self-healing hydrogels and control-release drug delivery system to carry out the spatial perspective. Last but not least, 3D printing of the bioink that contains patients’ stem cells is an important fabricating technique to combine smart polymeric materials and cell therapy to meet the personal aspect. Taken together, the development of smart polymeric materials plays an important role to incorporate all these three aspects for cell therapy and precision medicine.

## Data Availability

Not applicable

## Abbreviations

AM: Additive manufacturing
CDCs: Cardiac stem cells
CMC: Critical micelle concentration
ESCs: Embryonic stem cells
FSCs: Fibromatosis-derived stem cells
GelMA: Gelatin methacryloyl
hESC: human embryonic stem cell
iPSCs: induced pluripotent stem cells
Irgacure 2959: 2-Hydroxy-1-(4-(2-hydroxyethoxy)phenyl)-2-methyl-1-propano
LAP: Lithium phenyl-2,4,6-trimethylbenzoylphosphinate
LCST: Lower critical solution temperature
MSCs: Mesenchymal stem cells
PEO: Polyethylene oxide
PNiPAAm: Poly (N-isopropyl acrylamide)
PPO: Polypropylene oxide
PU NPs: Polyurethane nanoparticles
SLA: Stereolithography
SMAs: Shape memory alloys
SMMs: Shape memory materials
SMPs: Shape memory polymers
VA-086: 2,2’-Azobis[2-methyl-N-(2-hydroxyethyl)propionamide]
